# Tamoxifen for the treatment of polycystic liver disease: A case report

**DOI:** 10.1097/MD.0000000000026797

**Published:** 2021-08-13

**Authors:** Sophie E. Aapkes, Lucas H.P. Bernts, M. van den Berg, Ron T. Gansevoort, Joost P.H. Drenth

**Affiliations:** a Department Nephrology, University Medical Center, Groningen, The Netherlands; b Department Gastroenterology and Hepatology, Radboud University Medical Center, Nijmegen, The Netherlands; c Department Gynecology, University Medical Center, Groningen, The Netherlands.

**Keywords:** case-report, estrogen, hepatic cyst, polycystic liver disease, tamoxifen

## Abstract

**Rationale:**

Polycystic liver disease is a rare disease characterized by the growth of numerous cysts in the liver. The liver function remains well preserved, but liver volumes can grow very large, and some patients ultimately need a liver transplantation. Other treatment options are limited and there is an unmet need for new therapeutic options.

**Patient concerns:**

We describe a 59-year-old patient with pain in the abdomen, especially when bending forward. Five years ago, she was diagnosed with breast cancer and as an incidental finding a couple of large liver cysts were diagnosed, explaining her abdominal pain.

**Diagnosis:**

Polycystic liver disease with several large liver cysts.

**Interventions:**

The patient was treated with tamoxifen, an estrogen receptor modulator, as treatment for her hormone receptor positive breast cancer. One of the liver cysts was aspirated.

**Outcomes:**

In the 4.6 years after the start of tamoxifen treatment, 20 mg once daily, the volume of her liver cysts decreased remarkably. There was a reduction of combined cyst volume from 311 mL to 22 mL without percutaneous drainage.

**Lessons:**

Epidemiological as well as experimental evidence supports a pivotal role for estrogens as a driver for growth of polycystic livers. Estrogen antagonism has often been proposed as a therapeutic target, but supporting evidence is lacking in the literature. We hypothesize that the decrease in cyst size in this patient was caused by tamoxifen therapy, suggesting an in vivo antagonistic effect on cystic cholangiocytes. This is an important finding because tamoxifen could be a promising new treatment option for polycystic liver disease.

## 1 Introduction

Polycystic liver disease (PLD) is a rare disease characterized by growth of numerous cysts in the liver and hepatomegaly.^[1]^ While synthetic function remains preserved, the hepatomegaly may cause severe symptoms and a compromised quality of life, necessitating a liver transplantation.^[1,2]^ Somatostatin analogues are the only available drug option that reduce cyst growth, but the effect is relatively small (3%–8% reduction of liver volume after 6–12 months of treatment) and it is not uniformly effective.^[3]^ This highlights the need for new therapies. Epidemiological as well as experimental evidence points to an important role of estrogens as drivers of liver cyst growth.^[4–6]^ As such, the estrogen pathway may be an important therapeutic target for new medical therapies. Tamoxifen, a selective estrogen-receptor modulator (SERM), has been specifically suggested as an option.^[7,8]^

We describe a PLD patient with large liver cysts, who received tamoxifen as treatment for hormone receptor positive breast cancer. During tamoxifen treatment the volume of the liver cysts decreased remarkably. The patient gave informed consent for publication and because all interventions took part in the framework of clinical care, no ethics approval was needed.

## 2 Case report

**Figure 1:**
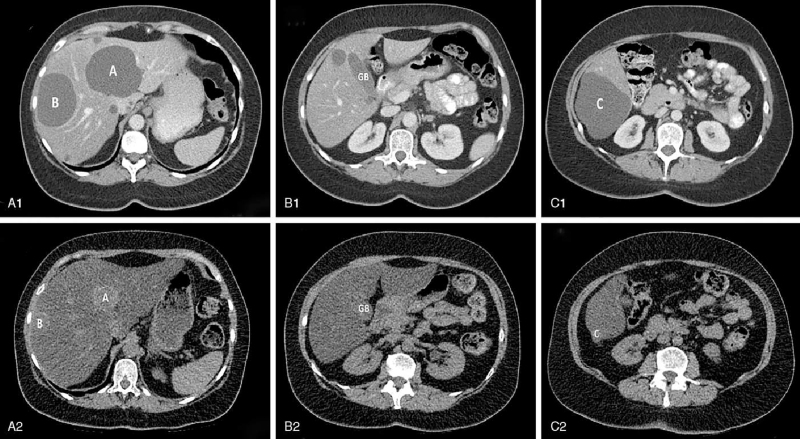
CT scans at start of tamoxifen, and 4.6 years later. 1A–C, Axial images of a contrast enhanced CT-scan of the liver (venous phase), performed just at the start of tamoxifen. Three large cysts in segment 4 (cyst B), 6 (cyst C), and 7 (cyst A) and multiple small cysts are present in the liver. The large, irregular cyst in segment 6 (cyst C) is either composed of multiple adjoining cysts or contains septations, caused by cyst bleeding. 2A–C, A low-dose CT-scan 4.6 years later. Due to pronounced hepatic steatosis, cysts appear relatively hyperintense. All cyst volumes were reduced, while only the cyst in segment 6 (cyst C) had been treated with drainage and sclerotherapy. The gallbladder (GB) has also been marked for reference on both scans.

A 59-year-old female PLD patient with autosomal-dominant polycystic liver disease developed breast cancer at the age of 54 years. Pathological analysis had shown a pT2(m)N0Mx, infiltrative lobular carcinoma, grade II, 3.8 cm, multifocal, estrogen-receptor positive (90%), progesterone receptor positive (90%), and HER2neu negative. She was treated with a mastectomy, adjuvant radiotherapy, and chemotherapy (docetaxel, doxorubicin, cyclophosphamide). Six months later, endocrine therapy with tamoxifen, 20 mg, once daily, was started and continued unchanged for 5 years. A CT-scan that was made at the start of tamoxifen demonstrated multiple large liver cysts (Fig. 1). The total liver volume assessed with manual segmentations was 1934 mL and cyst volumes calculated with the ellipsoid formula were 176, 198, and 135 mL (segments 4, 6, and 7, respectively).

**Figure 2:**
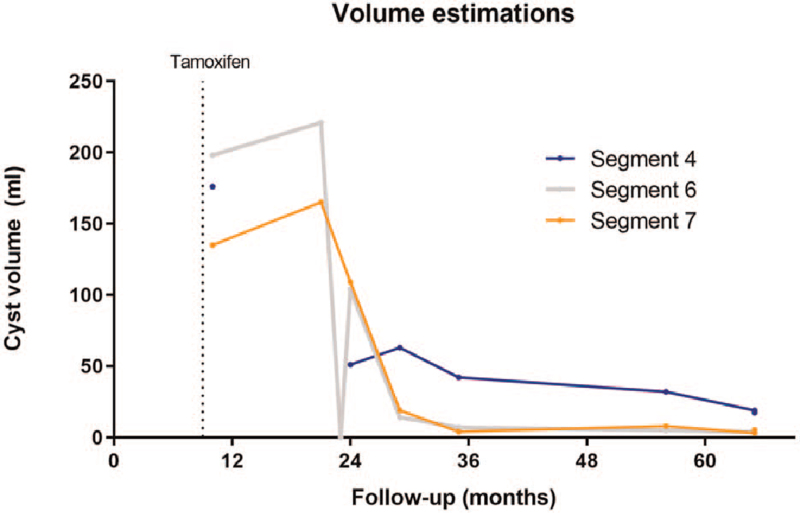
Cyst volumes from the 3 largest cysts during follow-up. Cyst volumes in milliliters on the Y-axis. Follow-up in months on the X-axis, starting with breast cancer diagnosis (t = 0). Dashed line represents start of tamoxifen therapy. Cyst volumes were estimated with the ellipsoid formula (π/6×width×depth×height). The first measurement was performed on a CT-scan, all follow-up measurements on ultrasounds. On the last follow-up visit, volumes were again measured using a CT-scan as well as an ultrasound on the same day. Volumes measured by both techniques correlated very well (both data points in graph). The cyst in segment 6 is the only cyst that has been treated with aspiration-sclerotherapy, whereas the other cysts decreased in volume without intervention. For the cyst in segment 4, the second value was missing.

One year after the start of tamoxifen use, the patient was referred because of symptoms that were ascribed to the presence of large liver cysts. She suffered from pain in the upper abdomen and right flank, abdominal fullness, early satiety, and difficulty bending forward. She had used estrogen-containing oral contraceptives for 25 years, up and until her breast cancer diagnosis. Hence, menopausal status was uncertain, as she had not menstruated for many years. Estrogen, follicle-stimulating hormone (FSH), luteinizing hormone (LH), or anti-mullerian hormone levels were not measured at this time. Cyst volume measurement on ultrasound showed further growth of the 3 large hepatic cysts compared with the first CT scan (221 and 165 mL, segments 6 and 7 respectively) (Fig. 2). The cyst in segment 4 was not measured at this time. Because of symptom localization and because it was accessible for percutaneous drainage, the cyst in segment 6 was treated with aspiration sclerotherapy, using 40 mL of 98% ethanol for 10 minutes. Other cysts were not treated.

At first follow-up, 4 weeks after the procedure, ultrasound was repeated. The treated cyst had refilled to an estimated 103 mL (original volume 221 mL), which is expected shortly after sclerotherapy.^[9]^ Remarkably, the other cysts had also decreased in volume without any intervention (Fig. 2). Subsequent follow-up with ultrasound showed further decline of all large cysts in the following 3 years. At last follow-up, the patient was 59 years and ultrasound and CT were performed. CT images showed liver steatosis, which could be a side effect tamoxifen.^[10]^ The total liver volume assessed with manual segmentations was 1869 mL and cyst volumes calculated with the ellipsoid formula were 19, 4, and 3 mL for segments 4, 6, and 7, respectively (Figs. 1 and 2). As such, there was a reduction of combined cyst volume from 311 mL to 22 mL without aspiration sclerotherapy in segments 4 and 7. At this point, the patient had still not menstruated, but also did not experience any symptoms of menopause. Although LH and FSH levels were inconclusive (FSH 13 units per liter [U/L], LH 11 U/L), the anti-mullerian hormone level (< 0.1 U/L) suggested that the patient had reached a postmenopausal status and the relatively low FSH and LH levels could be caused by the tamoxifen use.

## 3 Discussion

In this case report, we describe a female autosomal-dominant polycystic liver disease patient with large liver cysts, in whom the volume of multiple cysts declined remarkably over time. We hypothesize that this was caused by the anti-estrogenic effects of tamoxifen, in the standard dose of 20 mg daily.

### 3.1 The role of female hormones

Clinical as well as experimental evidence points to an important role for female hormones, including estrogen, in the growth of liver cysts.^[7,8]^ Supporting evidence includes, first, the fact that polycystic liver disease occurs more often in females, with a female-to-male ratio of 10 to 1, and that the disease is also more severe in women.^[11]^ Second, use of oral contraceptives and postmenopausal estrogen exposure has been shown to be associated with accelerated liver growth.^[4,5]^ Third, it has been shown that premenopausal women have faster liver growth compared with postmenopausal women. Spontaneous decrease in liver volume by 2% to 3% per year has been described previously, particularly in postmenopausal patients.^[4,12]^ This poses a limitation to our case report, as our patient stopped estrogen containing contraception shortly before the start of tamoxifen and presumably reached menopause during follow-up. However, as the effect on liver cyst size is much larger than expected in our case, we hypothesize that tamoxifen will have played a more important role.

These clinical findings are corroborated by experimental data. Estrogen receptors α and β have been found on cholangiocytes of cystic tissue from polycystic liver disease patients, while these receptors are absent in normal biliary structures of either polycystic liver disease patients or healthy controls.^[6]^ Addition of estradiol increases the proliferation of liver cyst-derived cholangiocytes in vitro.^[6]^ If estrogen receptor degraders such as fulvestrant are added, proliferation is significantly slowed.^[6]^ A possible role of progesterone in PLD remains to be elucidated.

### 3.2 Blocking the estrogen pathway

There is a clear need for new therapies to stop liver growth, especially in patients with severe PLD that do not benefit from somatostatin analogue treatment. The estrogen pathway in PLD may prove an important target for new therapies, as is recognized in the literature.^[7,8,13]^ However there are several challenges with anti-estrogenic therapy.

The most direct approach is treatment with drugs that block estrogen receptors, which are known as selective estrogen-receptor modulators (SERMs) or selective estrogen-receptor degraders (SERDs). A well-known SERM is tamoxifen, which is often used for hormone receptor positive breast cancer, and well-tolerated. However, SERMs can work either agonistic or antagonistic, dependent on the tissue that is studied.^[14]^ Tamoxifen, for example, acts as an antagonist in breast cancer cells, but as an agonist in bone and endometrial cells.^[15]^ This agonistic effect results in a beneficial effect on bone loss, but also in an increased risk of endometrial cancer. The effect of SERMs on cystic cholangiocytes is uncertain, but our case suggests a predominantly antagonistic effect for tamoxifen. This ambiguity is not seen with SERDs, because these dugs ensure protein degradation of estrogen-receptors.

A different approach is to halt systemic estrogen production, which can be achieved with gonadotropin-releasing hormone (GnRH)-antagonists or agonists. The antagonists work by directly inhibiting GnRH-receptors in the pituitary gland.^[16]^ Paradoxically, chronic treatment with the GnRH-agonists also leads to inhibition of estrogen production by pituitary desensitization. The subsequent fall in pituitary LH and FSH secretion suppresses ovarian follicular growth and ovulation, resulting in low levels of circulating estradiol and progesterone. The advantages of this approach are a smaller risk of severe adverse events compared with SERMs and SERDs and incidental reduction of progesterone levels, which may also play a role in hepatic cyst growth.

### 3.3 Limitations

In this case-report, and corroborated by literature, it seems that tamoxifen reduces the volume of hepatic cysts. However, there are some limitations. First, this patient was treated for her breast cancer with tamoxifen, but also with radiation and chemotherapy (docetaxel, doxorubicin, cyclophosphamide). Since these treatments affect especially fast-growing tissue, it could be that they slowed cyst growth. However, tamoxifen was started after cessation of these therapies, and the reduction in cyst volume took place about a year after the start of tamoxifen (Fig. 2). Second, the patient reached menopause during the 5-year treatment with tamoxifen. It is known from the literature that the liver cyst volume can decline after menopause,^[17]^ but that had not been reported to this extent.

### 3.4 Conclusion

We found that the 4.6 years of use of tamoxifen coincided with a remarkable volume reduction of hepatic cysts. There was a reduction of combined cyst volume from 311 mL to 22 mL, without aspiration sclerotherapy. Although we cannot fully rule out that menopause or any other source of bias played a role in the reduction of cyst volume, the decrease in cyst volume is much larger than expected. We hypothesize that the reduction in cyst size was caused by tamoxifen treatment, suggesting an in-vivo antagonistic effect on cystic cholangiocytes. In view of the need of new treatment options in PLD, this is an important finding because tamoxifen could be a promising therapy for PLD. We hope that our case may contribute to recognition of similar cases and further evaluation of this potential treatment option.

## Author contributions

**Conceptualization:** Sophie E. Aapkes, Lucas H.P. Bernts.

**Investigation:** Sophie E. Aapkes, Lucas H.P. Bernts.

**Resources:** M. van den Berg.

**Supervision:** Ron T. Gansevoort, Joost P.H. Drenth.

**Visualization:** Lucas H.P. Bernts.

**Writing – original draft:** Sophie E. Aapkes, Lucas H.P. Bernts.

**Writing – review & editing:** Sophie E. Aapkes.
